# Differential relationships of stress and HIV disclosure by gender: a person centered longitudinal study

**DOI:** 10.1186/s12889-021-10291-0

**Published:** 2021-02-02

**Authors:** Chengbo Zeng, Shan Qiao, Xiaoming Li, Xueying Yang, Zhiyong Shen, Yuejiao Zhou

**Affiliations:** 1 South Carolina SmartState Center for Healthcare Quality, Arnold School of Public Health, University of South Carolina, Columbia, SC USA; 2 UofSC Big Data Health Science Center, University of South Carolina, Columbia, SC USA; 3 Department of Health Promotion, Education, and Behavior, Arnold School of Public Health, University of South Carolina, Columbia, SC USA; 4 Guangxi Center for Disease Prevention and Control, Nanning, Guangxi China

**Keywords:** HIV, Disclosure, Stress, Gender, China

## Abstract

**Background:**

Existing literature mostly consider HIV disclosure as a static event and investigate its relationship with stress using a cross-sectional design. It is unclear about the dynamic changes of HIV disclosure levels (defined as the number of disclosure targets) and how stress may influence these changes. This study explored different disclosure levels using a person-centered longitudinal approach, examined whether stress could predict these disclosure levels, and investigated if this relationship differed by gender among people living with HIV (PLWH).

**Methods:**

Data were derived from a prospective cohort study conducted from November 2016 to January 2018 in Guangxi, China. Four hundred forty-four PLWH were included. Participants were assessed on perceived stress, sociodemographic characteristics, and number of HIV disclosure targets at baseline, 6-month, and 12-month follow-ups. Growth mixture modeling was used to characterize disclosure levels based on the changes of disclosure target number. Multinomial logistic regression was used to predict disclosure levels with baseline stress after adjusting for covariates. The interaction effect of stress by gender was examined. Adjusted odds ratio (*AOR*) with its 95% confidence interval were reported to show the strength of association.

**Results:**

Three levels of disclosure were characterized as “*Low levels of disclosure*” (Level One), “*Increased levels of disclosure*” (Level Two), and “*High levels of disclosure*” (Level Three). Accordingly, 355 (81.2%), 28 (6.4%), and 64 (12.4%) of PLWH were categorized respectively under low, increased, and high levels of disclosure. The interaction of baseline stress by gender was significant in differentiating Level One from Three (*AOR* = 0.85 [0.74 ~ 0.99]) while it was not significant between Level One and Two (*AOR* = 0.96 [0.81 ~ 1.15]). Compared to female, male PLWH with higher baseline stress had lower probability to have consistent high disclosure levels over time. PLWH who were married/cohabited had lower probability of being classified into consistent high levels of disclosure than low level (*AOR* = 0.43 [0.19 ~ 0.94]).

**Conclusions:**

There was gender difference in the relationship between stress and levels of HIV disclosure. To promote HIV disclosure, gender tailored interventions should be employed to help PLWH cope with stress.

**Supplementary Information:**

The online version contains supplementary material available at 10.1186/s12889-021-10291-0.

## Background

Despite that the global efforts on HIV prevention and control have significantly reduced HIV-related morbidity and mortality, the HIV epidemic in the world is still a heavy burden for public health [1]. According to the Joint United Nations Programme on HIV/AIDS (UNAIDS) report, there were about 38.0 million (31.6 ~ 44.5 million) people living with HIV (PLWH) and 690,000 AIDS-related deaths worldwide by the end of 2019 [1]. In China, the cases of HIV infection have been steadily increasing since 2015, with more than 1.25 million PLWH and an estimated of 80,000 (60,000 ~ 105,000) new diagnosed cases in 2018 [2]. More efforts are needed to prevent and control HIV epidemic.

Promoting HIV disclosure is an integral component of public health efforts to deter HIV transmission and improve linkage to and retention in HIV care [3]. For instance, Ding and colleagues found that HIV disclosure was positively associated with the access to healthcare resources in China [4]. HIV disclosure could benefit PLWH by enhancing the individual (e.g., medication adherence) [5–7], dyadic (e.g., condom use) [7–9], and social contextual (e.g., HIV awareness) outcomes [4, 10]. To promote HIV disclosure, it is important to investigate the HIV disclosure levels (e.g., number of disclosure targets) within a longitudinal frame because literature suggest that levels of HIV disclosure are not static but would change across time. For example, Knettel and colleagues found that among HIV-infected pregnant women who did not disclose their HIV status to anyone at the time of diagnosis, more than 40.0% of them disclosed to others by the 3 months postpartum [11].

Stress caused by the disease progression of HIV infection and consequence assessment of HIV disclosure could influence the actual disclosure behavior [12]. According to the disease progression theory of HIV disclosure, PLWH disclose their diagnosis when the disease progression results in hospitalizations and physical deterioration, which cannot be kept as a secret [3, 12]. As the disease progresses, the accumulative stress of keeping HIV infection as a secret will increase the need for evaluating the consequence of disclosure [12]. Based on the consequence theory, Serovich suggested that the association of disease progression with actual behavior of HIV disclosure might be moderated by consequence expectancy, which is defined as the consequences one anticipates from disclosure. The expectation of positive consequences, such as healthcare access and social support, resulting from HIV disclosure could help PLWH cope with stress and inspire their actual disclosure behavior. However, previous studies found an inconsistent relationship between stress and disclosure [3, 13, 14]. Deribe and colleagues demonstrated that stress related to advanced disease stage was positively associated with HIV disclosure but Lee and Rotheram-Borus found that there was no association between baseline stress and HIV disclosure over time [13, 14]. Most of these studies employed a cross-sectional design with limited ability to examine the causal relationship between stress and disclosure [13, 15, 16].

Existing studies found a gender difference in HIV disclosure and disclosure targets among PLWH. One study found that men were more likely to disclose their HIV status to friends and less likely to disclose to family members while the inverse result was found among women [17]. The possible reason might be that men and women may have different perceptions regarding the role of disclosure in coping with stress and obtaining support from different targets [18]. However, there is a dearth of studies investigating the gender difference in the relationship between stress and HIV disclosure. Investigating the gender difference in this relationship could inform the effective and tailored interventions to reduce stress and promote HIV disclosure among PLWH.

HIV disclosure could be a dynamic process and stress may be a potential correlate, but more evidence is needed to strengthen our understanding about the relationship between stress and HIV disclosure levels as well as the role of gender in this relationship from a longitudinal perspective. Employing person-centered approach and using data derived from a prospective cohort, the current study aimed to address the knowledge gaps by identifying different levels of HIV disclosure (number of disclosure targets) across time and testing whether perceived stress at baseline could predict these HIV disclosure levels. Given the gender difference in HIV disclosure, this study also tested if gender could moderate the association between baseline stress and levels of disclosure.

## Methods

### Study sites and participants

Data used in this study were derived from a prospective cohort study which was conducted from November 2016 to January 2018 in Guangxi, China. Guangxi was consistently ranked the third in the number of HIV case among 31 provinces in China from 2014 to 2018 [2]. In collaboration with the Guangxi CDC, we ranked all 17 cities and 75 rural counties in Guangxi in terms of number of reported HIV/AIDS cases. We selected the top two cities (urban centers) and top eight rural counties with the largest number of reported HIV/AIDS cases by 2014 as the study sites. All selected cities/counties agreed to participate.

The inclusion criteria for PLWH included: 1) at least 18 years of age; 2) a confirmed diagnosis of HIV; and 3) lived in Guangxi. The exclusion criteria were PLWH who had: 1) mental or physical inability to respond to assessment questions; 2) currently incarcerated or institutionalized for drug use or commercial sex; and 3) planned to permanently relocate outside of the province within a year. Based on our previous experience of recruitment in Guangxi, China, the response rate was higher than 95%. Four hundred and forty-six PLWH were recruited in the cohort study, and two participants were excluded from this study due to the missing data in gender. Hence, the final sample size in this study was 444.

### Data collection

Instruction for survey interviewers was developed in this study, and the interviewer-administered questionnaire was used for data collection. Medical staff or HIV case managers at the study sites referred potential participants to local team members. Local team members screened PLWH for eligibility and discussed with the prospective participants about the benefits and risks of the study and invited them to join. After obtaining their written informed content, the face-to-face interviews were conducted in private rooms of study sites. The interviewers read each question in the questionnaire, and the participants gave an oral response to the interviewer. By using this method, we could minimize the potential effect of varying degrees of literacy on participants’ ability to understand the items. Clarification would be provided by the interviewers as needed. Each participant received a gift (e.g., household items) equivalent to US$5.00 (1 USD ≈ 6.5 Chinese currency RMB at the time of the survey) at the completion of the interview. Data were collected at baseline, 6-month, and 12-month follow-ups. The study protocol was approved by the Institutional Review Boards at both University of South Carolina in the United States and Guangxi CDC in China.

### Variables and measurement

This section introduces the variables and measurements used in the current study. It covers detailed description about sociodemographic characteristics, number of HIV disclosure targets, and perceived stress. Supplementary 1 shows the questionnaire used in this study.

Participants provided information on their sociodemographic characteristics including age, gender (female or male), ethnicity (non-Han or Han), employment (unemployed or employed), level of education (illiteracy/primary school, or middle school, or above), monthly income in Chinese currency RMB (0 ~ 1999 or 2000 ~), marital status (married/cohabited or others), duration of HIV diagnosis, and antiretroviral therapy (ART) status (yes or no).

Participants reported their potential number of HIV disclosure targets (NHDT) in a list of 12 different social relationship, such as spouse, casual partner, parent, children, sibling, relatives, employer, and coworker. HIV disclosure in the current study was estimated using the awareness of the participants’ HIV status (0 = No/not applicable, 1 = Yes) among these 12 types of potential targets. The sum of the responses to 12 items ranging from 0 to 12 was calculated, with a higher score indicating a larger NHDT.

Perceived stress was assessed using the 14-item Perceived Stress Scale (PSS) [19]. The PSS measures global stress experienced or perceived during the past 30 days. Item samples were “how often have you been upset because of something that happened unexpectedly”, “how often have you felt nervous and ‘stressed’”, and “how often have you dealt successfully with irritating life hassles” [19]. Items were scored from 0 (“never”) to 4 (“very often”). The summed score ranging from 0 to 56 was used as the indicator of perceived stress, with a higher score indicating a greater level of stress. The 14-item PSS has been validated in the Chinese population [20]. The Cronbach’s alpha of the scale for the current study sample was 0.74 at baseline.

### Statistical analysis

First, descriptive statistics were reported on sociodemographic characteristics (e.g., age, gender), NHDT, and baseline stress. Median and interquartile range (IQR) were used to describe continuous variables (e.g., age, duration of HIV diagnosis, NHDT), and frequencies and percentages were used to describe categorical variables (e.g., gender, employment, level of education).

Second, a person-centered approach (i.e., growth mixture modeling [GMM]) was employed to identify different levels of HIV disclosure using NHDT across baseline, 6-month, and 12-month follow-ups [21]. Compared with existing studies which only calculated the average levels of HIV disclosure among PLWH across the time, GMM could classify PLWH into different latent subgroups with different levels of disclosure from a longitudinal perspective [22]. According to the standard procedure of GMM [22], a baseline, single-group model with linear change was specified. Then, successive models with increasing numbers of subgroup were fitted until the final model with appropriate number of subgroups were identified. Each model solution was replicated 20 times with beginning at random starting values.

The final number of subgroups was determined on the basis of model interpretation, size of estimated subgroup proportions and model fit indices including Akaike information criterion (AIC), Bayesian information criterion (BIC), adjusted BIC, entropy, *p*-values of likelihood ratio test (LRT) and bootstrap LRT (BLRT) [23, 24]. The level of HIV disclosure in each subgroup was interpreted and assessed whether it was supported by empirical evidence. Latent subgroups with less than 5% of the total sample were not considered due to the possibility of class over-extraction in the presence of non-normal data and poor generalizability [24]. Lower values on the information criteria (i.e., AIC, BIC, adjusted BIC) indicate better fitting models [21, 22]. The final model was selected if entropy was larger than 0.80 and the *p*-values of LRT and BLRT were insignificant. Missing values were handled using full information maximum likelihood method (FIML). Covariates (e.g., gender, employment, levels of education, marital status) were adjusted during the process of GMM.

Third, after obtaining the classification of HIV disclosure levels, bivariate analyses (i.e., Kruskal-Wallis test, Chi-square test, Fisher exact test) were conducted to identify the potential confounders in the relationship between stress and levels of HIV disclosure. Chi-square or Fisher exact tests were used to examine the bivariate relationship between categorical variables and different HIV disclosure levels while Kruskal-Wallis test was used for the relationship between continuous variable and HIV disclosure levels. Variables with *p*-values less than 0.25 were considered as potential confounders and were controlled in multivariate analysis.

Finally, multinomial logistic regression was conducted to predict levels of HIV disclosure using baseline stress as a predictor and adjusting for the potential confounders. The interaction of baseline stress by gender was also examined in the regression. Baseline stress was centered before the creation of interaction term. Simple slope analysis was performed to interpret the interaction. Adjusted odds ratio (AOR) with its 95% confidence interval (CI) were reported to show the strength of association. SAS software version 9.4 (SAS Institute, Inc., Cary, NC) was used to carry out the descriptive statistics, bivariate analyses, and multinomial logistic regression while Mplus version 7.0 (Muthen & Muthen, Los Angeles, CA) was used for GMM.

## Results

### Characteristics of the participants

Participants’ sociodemographic characteristics are shown in Table 1. The median (IQR) age of the participants were 42.0 (35.0 ~ 48.0) years old with a range from 18.0 to 67.0. More than half of the participants were male (302 [68.0%]), having at least middle school education (232 [52.7%]) and monthly income less than 2000 RMB (243 [54.9%]). Most of the PLWH were employed (340 [76.7%]) and receiving ART (398 [93.0%]).

**Table 1:** Descriptive statistics of study participants (*n* = 444)

Variables	Missing cases (%)	N (%)
**Total**	–	444 (100.0)
**Age (Years, median [IQR])**	27 (6.1)	42.0 (35.0 ~ 48.0)
**Duration of HIV diagnosis (Months, median [IQR])**	39 (8.8)	12.0 (0.0 ~ 24.0)
**Gender**	0 (0.0)	
Male		302 (68.0)
Female		142 (32.0)
**Employment**	1 (0.0)	
No		103 (23.3)
Yes		340 (76.7)
**Level of education**	4 (0.0)	
Illiteracy/primary school		208 (47.3)
Middle school or above		232 (52.7)
**Monthly income (RMB)**	1 (0.0)	
0 ~ 1999		243 (54.9)
2000 ~		200 (45.1)
**Marital status**	2 (0.0)	
Married/cohabited		342 (94.5)
Others		19 (5.3)
**ART initiation**	16 (3.6)	
Yes		398 (93.0)
No		30 (7.0)
**Ethnicity**	4 (0.0)	
Han		267 (60.7)
Non-Han		173 (39.3)
**NHDT** **(median [IQR])**		
Baseline (T_0_)	5 (0.0)	1.0 (1.0 ~ 2.0)
6-month (T_1_)	53 (11.9)	1.0 (1.0 ~ 3.0)
12-month (T_2_)	18 (4.1)	2.0 (1.0 ~ 3.0)
**Baseline perceived stress (median [IQR])**	1 (0.0)	38.0 (32.0 ~ 42.0)

Note: NHDT: Number of HIV disclosure targets

Attrition rates at 6-month and 12-month follow-ups were 11.9 and 4.1%, respectively. The median (IQR) NHDT at baseline, 6-month, and 12-month follow-ups were 1.0 (1.0 ~ 2.0), 1.0 (1.0 ~ 3.0), and 2.0 (1.0 ~ 3.0), respectively. The median score (IQR) of baseline stress was 38.0 (32.0 ~ 42.0).

### Growth mixture model of disclosure levels

The AIC, BIC, and adjusted BIC decreased with the numbers of disclosure levels increased (Table 2). As the *p*-values of LRT were significant until a four-level solution was found, there was no significant improvement in model fit between three-level and four-level solutions. However, due to the low proportion (1.6%) of one group in four-level solution, it was not considered as the appropriate solution. Among the remaining models (i.e., two- and three- level solutions), the three-level model had the lowest values of information criteria (AIC, BIC, and adjusted BIC). Additionally, its entropy was larger than 0.80. Given that the third-level solution also provided better interpretation of change in disclosure levels, it was selected as the final solution.

**Table 2:** Model fits (*n* = 437)

Models	*G*^*2*^*/LL*	*AIC*	*BIC*	*aBIC*	*Entropy*	*LMR*	*BLRT*
Baseline	− 1740.69	3519.44	3584.72	3533.95	–	–	–
2	− 1610.16	3266.31	3360.15	3287.16	0.99	< 0.001	< 0.001
**3**	**− 1538.30**	**3136.60**	**3259.00**	**3163.79**	**0.95**	**0.001**	**< 0.001**
4	− 1449.26	2972.52	3123.47	3006.06	0.95	0.116	< 0.001

Note: The missing data in variables of interest resulted in a sample of 437 in the final model

**Fig. 1:**
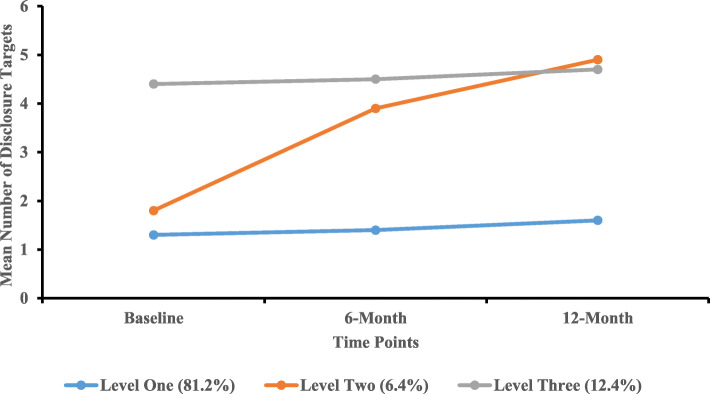
Different Levels of Disclosure among PLWH (*n* = 437)

Table 3 presents the proportion of each disclosure level for the three-level solution. For Level One (“*low levels of disclosure*”, 81.2%, 355/437), the median (IQR) NHDT across baseline, 6-month, and 12-month follow-ups were 1.0 (1.0 ~ 2.0), 1.0 (1.0 ~ 2.0), and 1.0 (1.0 ~ 2.0), respectively. The median (IQR) NHDT across baseline, 6-month, and 12-month follow-ups among PLWH in Level Two (“*increased levels of disclosure*”, 6.4%, 25/437) were 1.0 (1.0 ~ 3.0), 4.0 (3.0 ~ 5.0), and 4.5 (3.0 ~ 5.5), respectively. Finally, PLWH in Level Three (“*high levels of disclosure*”, 12.4%, 54/437) reported relatively large NHDT at each time point (baseline: 4.0 [4.0 ~ 5.0]; 6-month: 4.0 [4.0 ~ 5.0]; 12-month: 5.0 [4.0 ~ 5.0]). Based on the medians of NHDT, PLWH in Level Two had increased levels of disclosure while those in Levels One and Three had consistent disclosure levels. Figure 1 shows the number of disclosure targets for the three-level solution.

**Table 3:** Bivariate analyses between predictors and levels of HIV disclosure (*n* = 437)

Variables	Levels of HIV disclosure (%)			*p*-value
	Level One	Level Two	Level Three	
**Total**	355 (81.2)	28 (6.4)	54 (12.4)	–
**Age (Years, median [IQR])**	43.0 (36.0 ~ 48.0)	41.0 (33.0 ~ 46.0)	37.5 (32.0 ~ 44.0)	**< 0.01**^**a**^
**Duration of diagnosis (Month, median [IQR])**	12.0 (0.0 ~ 24.0)	12.0 (0.0 ~ 24.0)	12.0 (12.0 ~ 24.0)	0.21^a^
**Gender**				0.59^b^
Male	238 (67.0)	19 (67.9)	40 (74.1)	
Female	117 (33.0)	9 (32.1)	14 (25.9)	
**Employment**				0.12^b^
No	75 (21.1)	9 (32.1)	17 (31.5)	
Yes	280 (78.9)	19 (67.9)	37 (68.5)	
**Level of education**				**< 0.01**^**b**^
Illiteracy/primary school	181 (51.0)	7 (25.0)	19 (35.2)	
Middle school or above	174 (49.0)	21 (75.0)	35 (64.8)	
**Monthly income (RMB)**				0.47^b^
0 ~ 1999	191 (54.0)	18 (64.3)	32 (59.3)	
2000 ~	163 (46.0)	10 (35.7)	22 (40.7)	
**Marital status**				**< 0.01**^**b**^
Married/cohabited	66 (18.6)	3 (10.7)	22 (40.7)	
Others	289 (81.4)	25 (89.3)	32 (59.3)	
**ART initiation**				0.22^c^
Yes	321 (93.3)	21 (84.0)	49 (94.2)	
No	23 (6.7)	4 (16.0)	3 (5.8)	
**Ethnicity**				0.13^b^
Han	132 (37.6)	12 (42.9)	28 (51.9)	
Non-Han	219 (62.4)	16 (57.1)	26 (48.2)	
**Baseline perceived stress (median [IQR])**	38.0 (33.0 ~ 42.0)	36.0 (33.0 ~ 42.0)	36.5 (31.0 ~ 42.0)	0.69^a^
**NHDT** **(median [IQR])**				
Baseline (T_0_)	1.0 (1.0 ~ 2.0)	1.0 (1.0 ~ 3.0)	4.0 (4.0 ~ 5.0)	**< 0.01**^**a**^
6-month (T_1_)	1.0 (1.0 ~ 2.0)	4.0 (3.0 ~ 5.0)	4.0 (4.0 ~ 5.0)	**< 0.01**^**a**^
12-month (T_2_)	1.0 (1.0 ~ 2.0)	4.5 (3.0 ~ 5.5)	5.0 (4.0 ~ 5.0)	**< 0.01**^**a**^

Notes: Level One: “Low levels of disclosure”; Level Two: “Increased levels of disclosure”; Level Three: “High levels of disclosure”

^a^Kruskal-Wallis test

^b^Chi-square test

^c^Fisher exact test

NHDT: Number of HIV disclosure targets

### Associated factors of disclosure levels

Table 3 also presents the bivariate analyses of HIV disclosure levels. The results revealed that PLWH in different disclosure levels showed significant differences in their age, level of education, and marital status. The results of multinomial logistic regression predicting disclosure levels using variables with *p*-values less than 0.25 in bivariate analyses and perceived stress at baseline are shown in Table 4.

**Table 4:** Multinomial logistic regression of disclosure levels (*n* = 367)

Variables	*cOR (95%CI)*	*AOR (95%CI)*
*Level * Two vs. *Level One*		
**Gender (G)**		
Male	1.04 (0.46 ~ 2.36)	1.00 (0.36 ~ 2.77)
Female	Reference	
**Age**	0.96 (0.92 ~ 1.01)	0.96 (0.90 ~ 1.01)
**Duration of infection**	0.99 (0.95 ~ 1.03)	0.99 (0.95 ~ 1.04)
**Employment**		
Employed	1.77 (0.77 ~ 4.07)	2.23 (0.83 ~ 5.97)
Unemployed	Reference	
**Level of education**		
Illiteracy/primary school	0.32 (0.13 ~ 0.77)*	0.36 (0.12 ~ 1.06)
Middle school or above	Reference	
**Marital status**		
Married/cohabited	1.90 (0.56 ~ 6.49)	2.83 (0.59 ~ 13.56)
Others	Reference	
**ART initiation**		
Yes	0.38 (0.12 ~ 1.19)	0.37 (0.11 ~ 1.27)
No	Reference	
**Ethnicity**		
Han	0.80 (0.37 ~ 1.75)	0.65 (0.26 ~ 1.62)
Non-Han	Reference	
**Perceived stress (P)**	0.99 (0.93 ~ 1.05)	1.02 (0.87 ~ 1.18)
G*P	–	0.96 (0.81 ~ 1.15)
*Level Three* vs. *Level One*		
**Gender (G)**		
Male	1.40 (0.74 ~ 2.68)	1.30 (0.53 ~ 3.21)
Female	Reference	
**Age**	0.95 (0.92 ~ 0.98)*	0.96 (0.92 ~ 1.00)
**Duration of infection**	1.02 (0.99 ~ 1.05)	1.02 (0.99 ~ 1.05)
**Employment**		
Employed	1.72 (0.92 ~ 3.22)	1.22 (0.53 ~ 2.82)
Unemployed	Reference	
**Level of education**		
Illiteracy/primary school	0.52 (0.29 ~ 0.95)*	0.55 (0.25 ~ 1.20)
Middle school or above	Reference	
**Marital status**		
Married/cohabited	0.33 (0.18 ~ 0.61)*	0.43 (0.19 ~ 0.94)*
Others	Reference	
**ART initiation**		
Yes	1.17 (0.34 ~ 4.05)	1.25 (0.27 ~ 5.91)
No	Reference	
**Ethnicity**		
Han	0.56 (0.32 ~ 1.00)*	0.52 (0.25 ~ 1.07)
Non-Han	Reference	
**Perceived stress (P)**	0.99 (0.94 ~ 1.04)	1.12 (0.99 ~ 1.27)
G*P	**–**	0.85 (0.74 ~ 0.99)*

Notes: The missing data in variables of interest resulted in a sample of 367 in the multinomial logistic regression model

*cOR* crude odds ratio, *AOR* adjusted OR, G*P Interaction of perceived stress by gender, * *p *< 0.05

**Fig. 2:**
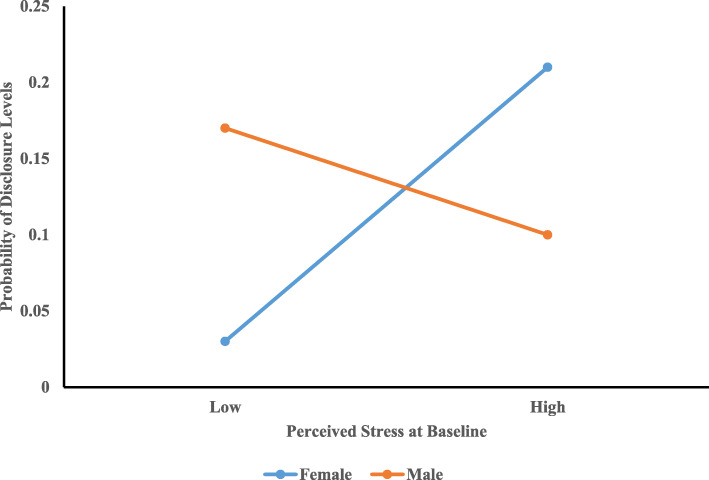
Interaction between Perceived Stress at Baseline and Gender in Predicting Levels of Disclosure between Level One and Level Three. Note: High and low are defined as one standard deviation above or below the mean, respectively

**Fig. 3:**
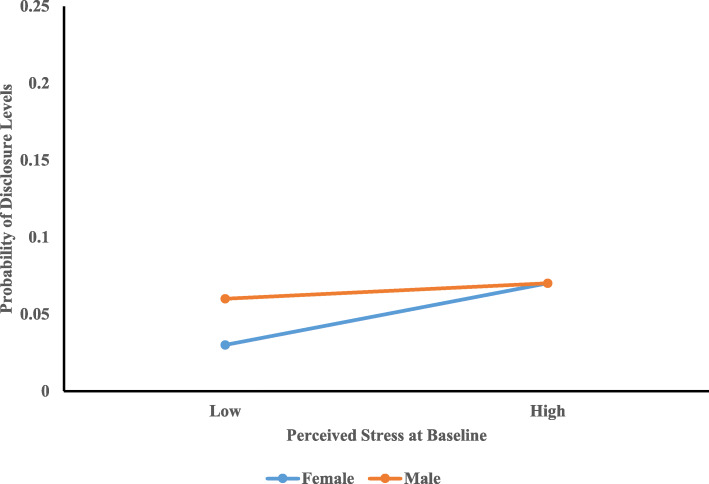
Interaction between Perceived Stress at Baseline and Gender in Predicting Levels of Disclosure between Level One and Level Two. Note: High and low are defined as one standard deviation above or below the mean, respectively

Multinomial logistic regression was conducted with adjusting for the variables with *p*-values less than 0.25 in bivariate analyses. PLWH in Level One was considered as the reference group in multinomial analysis. There was no significant relationship between perceived stress at baseline and levels of HIV disclosure. The interaction between gender and perceived stress at baseline was statistically significant (*AOR* = 0.85, 95%CI: 0.74 ~ 0.99) when comparing Level Three (“*high levels of disclosure*”) with Level One (“*low levels of disclosure*”) (Fig. 2). Compared to female PLWH, under the same levels of perceived stress at baseline, male PLWH had low probability of being classified into Level Three (“*high levels of disclosure*”). No significant interaction between gender and perceived stress at baseline (*AOR* = 0.96, 95%CI: 0.81 ~ 1.15) were found when comparing Level Two (“*increased levels of disclosure*”) with Level One (“*low levels of disclosure*”) (Fig. 3). Being married/cohabitated with partners decreased the probability of being classified into Level Three (“*high levels of disclosure*”) as compared to Level One (“*low levels of disclosure*”) (*AOR* = 0.43, 95%CI: 0.19 ~ 0.94).

## Discussion

Using data from a prospective cohort study, the current study employed a person-centered approach and identified three levels of HIV disclosure reflecting by different NHDT across time. Although there was no significant relationship between stress and levels of HIV disclosure among the overall sample, gender difference in this relationship was detected. Being married/cohabited with partners could significantly predict levels of HIV disclosure. To the best of our knowledge, this is one of the first studies using a person-centered approach to investigate different levels of HIV disclosure and examine the differential impacts of stress on HIV disclosure levels by gender from a longitudinal perspective.

Compared with previous studies on the patterns of HIV disclosure with a cross-sectional design [25–27], the current study used longitudinal data and confirmed that levels of HIV disclosure changed across the time, which was consistent with that of a previous study. Using data from a longitudinal study of men in San Francisco, Hays and colleagues found that HIV-infected men were most likely to disclose their serostatus to lovers and closest same-sex friends within one-year follow-up [28]. However, they did not investigate the changes of HIV disclosure levels. In our study, we found three HIV disclosure levels reflecting by the dynamic changes of NHDT over time with most participants reported consistently low levels of HIV disclosure. Additionally, we found that nearly 20.0% of the PLWH reported consistently large NHDT or increased NHDT acrosstime, and the maximum NHDT was closed to five. These findings could advance our understanding about the changes of HIV disclosure and call for future studies to investigate their correlates, which could inform targeted public health interventions and promote HIV disclosure among PLWH effectively.

Even though stress did not predict levels of HIV disclosure directly, there was a gender difference in this association. The significant interaction effect of perceived stress by gender on levels of HIV disclosure indicated that with the same levels of stress, males and females might have different attitudes towards disclosing their HIV serostatus and selecting disclosure targets to seek help, which might result in different levels of disclosure. Compared to male counterparts, female perceiving high levels of stress at baseline were more likely to be classified into Level Three and reported consistently large NHDT over time. The possible explanation for this finding was that females were more likely than males to seek help and cope with stress through HIV disclosure [29, 30]. For instance, Xiao and colleagues found that female PLWH in Guangxi were more in need for social support and advice on coping strategies than male PLWH [29]. Thus, they might seek support and coping strategies through disclosing their HIV serostatus. The stress by gender interaction effect between Levels One (“*low levels of disclosure*”) and Two (“*increased levels of disclosure*”) was non-significant possibly due to the small sample size in Level Two, which might limit the statistical power to detect significant effects of interaction or other covariates of HIV disclosure levels. Future studies with large sample sizes are needed to investigate the relationship between stress and increased levels of disclosure as well as the role of gender in this relationship.

Interestingly, PLWH who married/cohabited with partners reported consistently small NHDT from baseline to follow-ups. For PLWH who married/cohabited with partners, they only disclosed their HIV status to the significant others (e.g., spouse and stable sexual partners) who could provide them with sufficient support [31, 32]. Keeping their HIV serostatus to significant others and avoiding unnecessary disclosure could protect themselves from unintended consequences. For PLWH who were single, divorced or widowed, as they were more likely to disclose their HIV infection to more targets, future efforts are still needed to improve the quality of HIV disclosure and ensure beneficial consequences of the disclosure.

To promote HIV disclosure, structural and individual interventions are needed. At the structural levels, the effort on promoting HIV-related health education could improve the awareness of HIV, reduce the discrimination against PLWH, and intervene their perceived stress [33, 34]. For PLWH, efforts are also needed to improve their education and skill of HIV disclosure at both community and clinical settings [18, 35]. In particular, these efforts should help PLWH identify the potential disclosure targets and teach them how to conduct HIV disclosure and avoid unintended consequences. At the individual level, resilience-based mental health interventions should be implemented to help PLWH cope with stress. Additionally, improving consequence assessment and self-efficacy for disclosure among PLWH could elevate their confidence and motivation to use HIV disclosure as a means to cope with stress [35]. In terms of the implementation of HIV disclosure, individual interventions are also needed to improve PLWH’s disclosure strategies, such as communication skills and ability to manage negative reactions [35]. All of these interventions should be tailored to gender and marital status.

Although this study was innovative in investigating different levels of HIV disclosure and their correlates from a longitudinal perspective, there are some limitations needed to acknowledge. First, self-report number of disclosure targets was employed in the current study, and self-report bias might exist. Second, the number of disclosure targets was estimated using the awareness of the participants’ HIV infection by potential targets although previous study demonstrated that this awareness was closely related to HIV disclosure [36]. Additionally, we treated all targets equally in the construction of the “levels of disclosure” but PLWH might weight different disclosure targets differently in the process of disclosure decision-making. As not all potential targets were applicable to each participant, the number of disclosure targets used in this study might be inaccurate. Furthermore, we did not differentiate whether the HIV disclosure was intended or unintended, which would influence the relationship between stress and disclosure. Third, the current study considered stress as a predictor of HIV disclosure, but disclosure might also influence levels of perceived stress in a long-term. Future studies are needed to investigate the bidirectional relationship between stress and HIV disclosure among PLWH. Fourth, the small sample size in Level Two and Three might limit the statistical power to detect the significant effects of variables of interest on different HIV disclosure levels. Future research with large sample sizes is needed to validate the results from the current study. Finally, the participants were representative of the PLWH in the current study sites, but cautions should be given when generalizing the findings from this study to other rural/urban residents in China as well as other countries.

## Conclusion

The results of this study shed light on the dynamic changes of HIV disclosure levels across time and how stress predicted these changes among PLWH. There was gender difference in the relationship between stress and levels of HIV disclosure. To promote HIV disclosure, structural interventions are needed to improve health education of HIV disclosure and help PLWH identify potential disclosure targets at both community and clinical settings. Tailored interventions based on individuals’ gender are needed to improve the stress management, consequence assessment, self-efficacy, and disclosure skills among PLWH.

## Supplementary Information

**Additional file 1.** Questionnaire includes all variables used in this study.

## Data Availability

The datasets generated and/or analyzed in the current study are available from the corresponding author on reasonable request.

## Abbreviations

AIC: Akaike information criterion
AIDS: Acquired immunodeficiency syndrome
ART: Antiretroviral therapy
BIC: Bayesian information criterion
BLRT: Bootstrap likelihood ratio test
CDC: Center for disease control and prevention
FIML: Full information maximum likelihood method
GMM: Growth mixture modeling
HIV: Human immunodeficiency virus
LRT: Likelihood ratio test
NHDT: Number of HIV disclosure targets
OR: Odds ratio
PLWH: People living with HIV
PSS: Perceived stress scale
SD: Standard deviation
