# Supplementary material for: Differential relationships of stress and HIV disclosure by gender: a person centered longitudinal study
Source: BMC Public Health. 2021 Feb 2;21:263. doi: 10.1186/s12889-021-10291-0 (PMC7852186; doi:10.1186/s12889-021-10291-0)
Supplement: Supplementary file 1 — Additional file 1. Questionnaire includes all variables used in this study. [file 12889_2021_10291_MOESM1_ESM.docx]

**Section 1: Sociodemographic characteristics**

**Q101. Years of age: _______**

**Q102. Gender:**

1. Male 2. Female

**Q103. Ethnicity:**

1. Han 2. Zhuang 3. Miao 4. Others: **_______**

**Q104. Marital status:**

1. Unmarried 2. Cohabitating 3. Married/remarried

4. Separated 5. Divorced 6. Windowed

**Q105. The highest education level:**

1. Illiteracy 2. Primary school 3. Middle school

4. High school 5. College or above

**Q106. Monthly household income:**

1. 0 ~ 999 RMB 2. 1,000 ~ 1,999 RMB 3. 2,000 ~ 2,999 RMB

4. 3,000 ~ 3,999 RMB 5. 4,000 ~ 4,999 RMB 6. 5,000 ~ RMB.

**Q107. Employment status:**

1. Unemployed 2. Part-time 3. Full-time

**Q107. Date of HIV diagnosis:** **_______** Year **_______** Month

**Q108. Whether currently receiving ART:**

1. Yes 2. No

**Section 2: HIV disclosure matrix**

This section would ask you whether these following targets know your HIV infection status. For each target, please indicate your response by placing an “X” to the relevant options.

| **Targets** | **Yes** | **No** | **Not applicable** |
| --- | --- | --- | --- |
| Spouse |  |  |  |
| Causal partner |  |  |  |
| Father |  |  |  |
| Mother |  |  |  |
| Children under 18 years old |  |  |  |
| Children older than 18 years old |  |  |  |
| Brothers |  |  |  |
| Sisters |  |  |  |
| Other relatives |  |  |  |
| Friends |  |  |  |
| Employer |  |  |  |
| Coworkers |  |  |  |

**Section 3: Perceived stress**

The questions in this scale ask you about your feelings and thoughts during the last month. In each case, please indicate your response by placing an “X” to represent HOW OFTEN you felt or thought a certain way.

| **Items** | **Never** | **Almost never** | **Sometimes** | **Fairly often** | **Very often** |
| --- | --- | --- | --- | --- | --- |
| How often have you been upset because of something that happened unexpectedly? |  |  |  |  |  |
| How often have you felt that you were unable to control important things in your life? |  |  |  |  |  |
| How often have you felt nervous and “stressed”? |  |  |  |  |  |
| How often have you dealt successfully with irritating life hassles? |  |  |  |  |  |
| How often have you felt that you were effectively coping with important changes that were occurring in your life? |  |  |  |  |  |
| How often have you felt confident about your ability to handle your personal problems? |  |  |  |  |  |
| How often have you felt that things were going your way? |  |  |  |  |  |
| How often have you found that you could not cope with all the things that you had to do? |  |  |  |  |  |
| How often have you been able to control irritations in your life? |  |  |  |  |  |
| How often have you felt that you were on top of things? |  |  |  |  |  |
| How often have you been angered because of things that happened that were outside of your control? |  |  |  |  |  |
| How often have you found yourself thinking about things that you have to accomplish? |  |  |  |  |  |
| How often have you been able to control the way you spend your time? |  |  |  |  |  |
| How often have you felt difficulties were piling up so high that you could not overcome them? |  |  |  |  |  |
